# Inflammatory marker testing in primary care in the year before Hodgkin lymphoma diagnosis: a UK population-based case–control study in patients aged ≤50 years

**DOI:** 10.3399/BJGP.2021.0617

**Published:** 2022-07-12

**Authors:** Meena Rafiq, Gary Abel, Cristina Renzi, Georgios Lyratzopoulos

**Affiliations:** Institute of Epidemiology and Health Care, UCL, London.; University of Exeter Medical School, Exeter.; Institute of Epidemiology and Health Care, UCL, London.; Institute of Epidemiology and Health Care, UCL, London.

**Keywords:** blood tests, diagnostic time window, general practice, Hodgkin lymphoma, inflammatory markers

## Abstract

**Background:**

Proinflammatory conditions are associated with increased risk of Hodgkin lymphoma, although the neoplastic process *per se* often induces an inflammatory response.

**Aim:**

To examine pre-diagnostic inflammatory marker test use to identify changes that may define a ‘diagnostic window’ for potential earlier diagnosis.

**Design and setting:**

This was a matched case–control study in UK primary care using Clinical Practice Research Datalink data (2002–2016).

**Method:**

Primary care inflammatory marker test use and related findings were analysed in 839 Hodgkin lymphoma patients and 5035 controls in the year pre-diagnosis. Poisson regression models were used to calculate monthly testing rates to examine changes over time in test use. Longitudinal trends in test results and the presence/absence of ‘red-flag’ symptoms were examined.

**Results:**

In patients with Hodgkin lymphoma, 70.8% (594/839) had an inflammatory marker test in the year pre-diagnosis versus 16.2% (816/5035) of controls (odds ratio 13.7, 95% CI = 11.4 to 16.5, *P*<0.001). The rate of inflammatory marker testing and mean levels of certain inflammatory marker results increased progressively during the year pre-diagnosis in Hodgkin lymphoma patients while remaining stable in controls. Among patients with Hodgkin lymphoma with a pre-diagnostic test, two-thirds (69.5%, 413/594) had an abnormal result and, among these, 42.6% (176/413) had no other ‘red-flag’ presenting symptom/sign.

**Conclusion:**

Increases in inflammatory marker requests and abnormal results occur in many patients with Hodgkin lymphoma several months pre-diagnosis, suggesting this period should be excluded in aetiological studies examining inflammation in Hodgkin lymphoma development, and that a diagnostic time window of appreciable length exists in many patients with Hodgkin lymphoma, many of whom have no other red-flag features.

## INTRODUCTION

The diagnosis of Hodgkin lymphoma in primary care is challenging and often delayed. This reflects its rarity, its predominance among young patients where cancer incidence is low, and the fact that fewer than a third of patients present with ‘red-flag’ symptoms (unexplained lymphadenopathy or lumps) prompting urgent referral.^1^ Most patients with Hodgkin lymphoma present with non-specific symptoms, such as fatigue, abnormal sweating, and pruritus, which have a broad range of differential diagnoses including many benign conditions frequently encountered in patients consulting in primary care.^2^ New approaches to improving the diagnosis are therefore needed.^3^^,^^4^

Conditions associated with chronic inflammation represent risk factors for developing Hodgkin lymphoma.^4^^–^^10^ Related to this, raised inflammatory markers have been associated with increased risk of Hodgkin lymphoma.^11^ However, the Hodgkin lymphoma disease process itself could also be associated with an inflammatory response;^12^^,^^13^ although this may introduce reverse causality (‘protopathic’) bias in aetiological studies examining the role of inflammation in Hodgkin lymphoma risk, it could also represent an early marker of as-yet-undiagnosed Hodgkin lymphoma, providing potential opportunities for more timely diagnosis.

The concept of a ‘diagnostic time window’ has been proposed to denote the pre-diagnostic period during which healthcare seeking and diagnostic activity increase from baseline. This represents the longest possible period during which the time-to-diagnosis could in principle be expedited in some patients.^14^^–^^16^ For aetiological studies, the length of the diagnostic window also defines the period during which reverse causality bias could occur when estimating causal associations; and the minimum pre-diagnostic period to be excluded from follow-up for this reason.^16^ Information about primary care blood test use has previously been used to estimate the length of such diagnostic windows for cancer sites other than Hodgkin lymphoma.^14^^,^^17^^,^^18^ Raised inflammatory markers are predictive of Hodgkin lymphoma risk,^11^ but when such abnormalities occur is unclear. Pottegård *et al* suggest that a 6-month lag-period should be applied in studies aiming to establish aetiological associations between exposure to a drug and risk of developing cancer, to avoid reverse causality from increased prescribing in the lead up to cancer diagnosis.^16^ Examining associations between inflammatory markers and risk of developing Hodgkin lymphoma is subject to similar concerns but the length of equivalent lag-periods to be applied is unknown.

Given the above background, this study aimed to examine associations between primary care inflammatory marker blood test use/findings and subsequent Hodgkin lymphoma diagnosis, and timing of changes in inflammatory markers pre-diagnosis.

**Table:** How this fits in

Understanding the timing of the inflammatory response in Hodgkin lymphoma may help identify opportunities for earlier diagnosis. In patients with Hodgkin lymphoma presenting to UK general practice, greater than expected and increasing use of inflammatory marker tests in the year before diagnosis were observed; two-thirds of patients with Hodgkin lymphoma who were tested for inflammatory markers had abnormal results, with almost half of patients in this group having no other recorded red-flag feature beyond their abnormal result. These findings provide proof of concept about the presence of a ‘diagnostic window’ during which Hodgkin lymphoma diagnosis could be expedited in at least some patients. Given the challenges of timely diagnosis in patients with Hodgkin lymphoma, inflammatory marker testing could help to expedite the diagnosis in those presenting with non-specific symptoms if supported and utilised by future advances in diagnostic technology.

## METHOD

### Data sources

A matched case–control study was undertaken using linked data from the UK Clinical Practice Research Datalink (CPRD) between 1 January 2002 and 31 July 2016. CPRD is a primary care electronic health record database containing anonymised information from GP consultations covering approximately 9% of all UK practices (in 2013).^19^ Coded information on diagnoses, GP laboratory results, and demographics are available. CPRD data were supplemented by linkage to Hospital Episode Statistics (HES) data (Set 13) for identification of Hodgkin lymphoma diagnoses coded in ICD-10 (International Classification of Diseases, 10th revision) for patients registered in England and Index of Multiple Deprivation quintile to provide data on socioeconomic status.

### Study population

Hodgkin lymphoma has a bimodal age-specific incidence pattern with peaks in younger and older adults, and likely different histological subtypes and aetiological processes in each group.^9^^,^^20^ Individuals aged ≤50 years actively registered with a CPRD practice for more than a year with ‘up-to-standard’ data for research purposes during the study period were eligible for inclusion. This age group is where the majority of Hodgkin lymphoma cases occur and where diagnostic difficulty is likely greater owing to malignant disease being rare and less often considered in younger patients.^21^ Patients were excluded if they had a previous diagnosis of Hodgkin lymphoma, or if the diagnosis was made within 1 year of registering with their practice.^22^ Patients in the case group were individuals defined as having a new diagnosis of Hodgkin lymphoma in either CPRD or HES between 1 January 2003 and 31 July 2016 (see Supplementary Table S1 for code lists), with the earliest recorded date of diagnosis taken as the index date. Six controls were individually matched to each person in the case group based on sex and age at index date (plus or minus 1 year of age) using concurrent matching.^23^ Each control was selected at random using a random number generator from the pool of eligible matches for each person in the case group. An index date was assigned to each control participant corresponding to the diagnosis date of their matched case participant. Data were analysed on all participants for 12 months before the index date.

### Defining inflammatory marker blood tests

Six common inflammatory markers were selected: erythrocyte sedimentation rate (ESR), C-reactive protein (CRP), plasma viscosity (PV), platelet count, ferritin concentration (whose values all increase as part of the inflammatory response), and albumin (whose values decrease during the inflammatory response). All such tests during the 12 months pre-diagnosis/index date were included. Data were collected for test date, the total number of tests per patient during the 12-month period, and the result (classified as normal or abnormal based on standard laboratory reference ranges).

Where units of measurement varied, the most frequently used units and reference ranges were identified, and where possible values were converted accordingly, with biologically implausible values excluded. For repeat tests (of the same kind) on the same day only one was counted to prevent duplicates and the mean value of the results from that day was used.

### Statistical analysis

Conditional logistic regression models were used to compare baseline characteristics of the study population and examine associations between both inflammatory marker test use (any test versus none) and abnormal results (any versus none) with Hodgkin lymphoma diagnosis in the following year, in the case and control groups. When considering all inflammatory marker tests together, the number of test requests and number of abnormal results were treated as ordinal variables in the final model (that is, 0,1, ≥2 requests per patient and 1, 2, ≥3 abnormal results across different tests in the year pre-diagnosis/index date). These paramaterisations improved model fit compared with use of respective binary (yes/no) variables and were deemed more clinically informative as having two or more tests indicates potential follow-up testing, and abnormal results across multiple different tests may increase the likelihood of disease. The proportion of patients who received a test request from their GP was also calculated for sequential 3-month time periods in the year before diagnosis/index date.

Mixed-effects Poisson regression analyses were used to examine time trends in GP inflammatory marker request rates. The total number of test requests per patient was modelled for each of the 12 months before Hodgkin lymphoma diagnosis in the case and control groups, including a random intercept for matching set given the matched study design. Testing rate ratios (RRs) were used to compare monthly request rates in patients with Hodgkin lymphoma compared with 1) their baseline rate, at 12 months pre-diagnosis and 2) the corresponding rate in controls at synchronous time points to identify the month at which the RR becomes significantly greater for the case participants than for the control participants, and to estimate the maximum diagnostic window length. The above analyses were repeated separately for each of the six inflammatory marker tests.

Among individuals in the Hodgkin lymphoma case group only, the timing of first inflammatory marker test events in the year before diagnosis was examined, by comparing ‘early’ (defined as 3–12 months before diagnosis) to ‘late’ (<3 months before diagnosis) tests, and estimating the proportion with an ‘early’ abnormal result versus exclusively ‘late’ abnormal results. Further, as a supplementary analysis, patient information on the presence of consultations with recorded ‘red-flag’ symptoms for Hodgkin lymphoma in the year preceding diagnosis (lymphadenopathy/lumps, night sweats, and weight loss^24^ — see Supplementary Table S2 for code lists) was also examined. This was used to explore how often inflammatory marker tests and abnormal findings occurred in patients with Hodgkin lymphoma without ‘red-flag’ symptoms to estimate the proportion of patients with Hodgkin lymphoma in which abnormal findings could be particularly useful. This was done by cross-tabulating red-flag symptom status (yes/no) by 1) inflammatory marker test use status, 2) abnormal inflammatory test status, and 3) ‘early’ abnormal inflammatory test status. Analyses were performed using Stata (version 16).

## RESULTS

In total, 839 people with Hodgkin lymphoma were matched to 5035 controls (Supplementary Figure S1). In the case group 46.9% (394/839) were identified using CPRD alone, 9.0% (76/839) were identified using HES alone, and 43.9% (369/839) were identified in both datasets. In addition to age and sex (matching variables), the participants in the case and control groups also had comparable socioeconomic status (Supplementary Table S3).

In the year before diagnosis 70.8% (594/839) of patients with Hodgkin lymphoma had at least one of the six examined inflammatory marker tests (CRP, ESR, PV, platelets, ferritin, or albumin) compared with 16.2% (816/5035) of controls (*P*<0.001) and tested patients had 14-fold greater odds of Hodgkin lymphoma diagnosis compared with controls (odds ratio 13.7, 95% confidence interval [CI] = 11.4 to 16.5, *P*<0.001). The odds of Hodgkin lymphoma diagnosis increased with increasing number of tests (*P*<0.001, Table 1). Similar increases were observed when considering each inflammatory marker test individually (Table 1). Among the 594 patients with Hodgkin lymphoma who had a test in the year pre-diagnosis, 41.6% (247/594) had an ‘early’ test (requested ≥3 months before diagnosis) and one in five had an ‘early’ abnormal result (Supplementary Table S4).

**Table 1.** Association between Hodgkin lymphoma and inflammatory marker blood tests in the year preceding diagnosis/index date

**Blood test and characteristics**	**Patients with Hodgkin lymphoma (*n*= 839)**	**Controls (*n* = 5035)**	**Absolute difference^a^**	**Odds ratio (95% CI)**	***P*-value^b^**
**Any inflammatory marker test**					
Tested in last year,^c^ *n* (%)	594 (70.8)	816 (16.2)	55.0	13.7 (11.4 to 16.5)	<0.001
Number of tests, *n* (%)					
1	43 (5.1)	167 (3.3)	1.8	4.66 (3.21 to 6.75)	<0.001
≥2	551 (65.7)	649 (12.9)	52.8	16.49 (13.57 to 20.04)	
Number of tests, median (range, IQR)	3 (0–33, 5)	0 (0–24, 0)	3.0	—	—
Number of different test types with an abnormal result,^d^ *n* (%)	594	816			<0.001
1	167 (28.1)	119 (14.6)	13.5	15.7 (11.7 to 21.1)	
2	135 (22.7)	24 (2.9)	19.8	80.7 (45.0 to 145.0)	
≥3	111 (18.7)	3 (0.4)	18.3	484.0 (146.7 to 1596.5)	

**Platelet**					
Tested in last year,^c^ *n* (%)	578 (68.9)	742 (14.7)	54.2	14.1 (11.7 to 17.0)	<0.001
Had abnormal result,^d^ *n* (%)	198 (34.3)	59 (8.0)	26.3	8.07 (5.02 to 12.99)	<0.001
Value, × 10^9^/l, mean (SD)	354.2 (143.2)	267.6 (74.8)	86.6	—	<0.001

**Albumin**					
Tested in last year,^c^ *n* (%)	452 (53.9)	542 (10.8)	43.1	11.1 (9.2 to 13.3)	<0.001
Had abnormal result,^d^ *n* (%)	95 (21.0)	36 (6.6)	14.4	3.16 (1.79 to 5.59)	<0.001
Value, g/l, mean (SD)	39.8 (6.0)	43.3 (4.5)	−3.5	—	<0.001

**ESR**					
Tested in last year,^c^ *n* (%)	361 (43.0)	213 (4.2)	38.8	17.7 (14.2 to 22.1)	<0.001
Had abnormal result,^d^ *n* (%)	266 (73.7)	78 (36.6)	37.1	6.37 (2.85 to 14.27)	<0.001
Value, mm/h, mean (SD)	39.0 (32.0)	11.4 (13.0)	27.6	—	<0.001

**CRP**					
Tested in last year,^c^ *n* (%)	320 (38.1)	191 (3.8)	34.3	17.8 (14.1 to 22.4)	<0.001
Had abnormal result,^d^ *n* (%)	217 (67.8)	32 (16.8)	51.0	50.0 (6.9 to 364.2)	<0.001
Value, mg/l, mean (SD)	49.9 (57.7)	7.9 (16.9)	42.0	—	<0.001

**PV**					
Tested in last year,^c^ *n* (%)	51 (6.1)	28 (0.6)	5.5	11.0 (6.9 to 17.4)	<0.001
Had abnormal result,^d^ *n* (%)	39 (76.5)	10 (35.7)	40.8	—	—
Value, mPa/s, mean (SD)	1.9 (0.2)	1.7 (0.1)	0.2	—	<0.001

**Ferritin**					
Tested in last year,^c^ *n* (%)	113 (13.5)	160 (3.2)	10.3	4.97 (3.82 to 6.45)	<0.001
Had abnormal result (high),^d^ *n* (%)	30 (26.5)	5 (3.1)	23.4	3.00 (0.31 to 28.84)	0.34
Value, µg/l, mean (SD)	230.5 (296.4)	64.9 (95.4)	165.6	—	<0.001

a *Data are percentages for variables presented with* n *(%).*

b P*-value from likelihood-ratio test from regression models (separate for each test) where case status was the outcome and each inflammatory marker test related variable was the exposure or from t-test for comparison of means.*

c *Twelve months pre-diagnosis date in the case participants or 12 months pre-index date in control participants.*

d *Percentage of all patients who had the test. CI = confidence interval. CRP = C-reactive protein. ESR = erythrocyte sedimentation rate. IQR = interquartile range. PV = plasma viscosity.*

Among tested patients, inflammatory markers were also more often abnormal in the year preceding diagnosis or index date in patients with Hodgkin lymphoma (69.5%, 413/594) compared with controls (17.9%, 146/816) (*P*<0.001, Table 1). A greater number of abnormal test results in the year pre-diagnosis (across different types of inflammatory markers) were also associated with greater odds of Hodgkin lymphoma (*P*<0.001, Table 1).

### Proportion of patients tested over time

**Figure 1.**
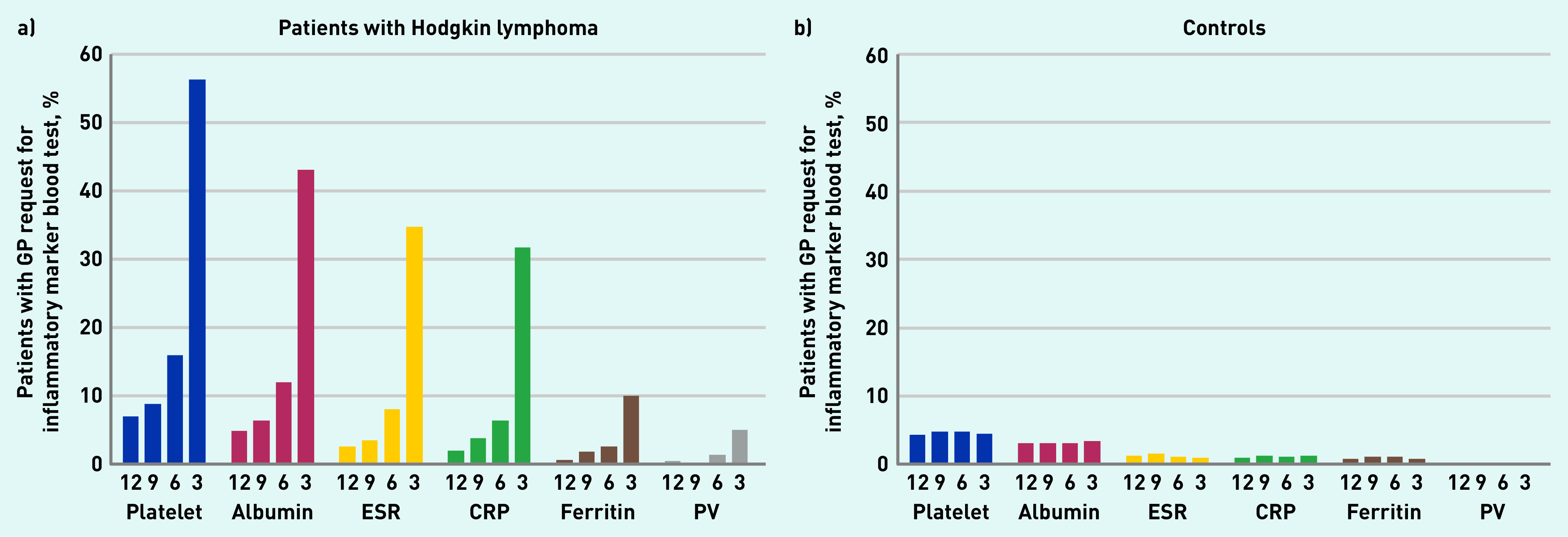
*Proportion of patients with a GP request for inflammatory marker blood test: for sequential 3-month periods in the year before diagnosis/index date in a) patients with Hodgkin lymphoma and b) controls. Numbers underneath bars represent months before diagnosis/index date. CRP = C-reactive protein. ESR = erythrocyte sedimentation rate. PV = plasma viscosity.*

The proportion of patients having at least one inflammatory marker test was consistently higher in patients with Hodgkin lymphoma than controls for each sequential 3-month time period in the year pre-diagnosis, with similar patterns across all six tests (Figure 1). The most notable increase in test use among patients with Hodgkin lymphoma was seen for platelet count (part of full blood count) and albumin concentration (part of liver function tests), the proportion of patients with Hodgkin lymphoma having these tests gradually increasing from <10% at 10–12 months pre-diagnosis to 56.3% (472/839) and 43.0% (361/839) in the 3 months immediately preceding diagnosis, respectively, while remaining stable at <10% in controls (Figure 1).

### Test request rates over time

**Figure 2.**
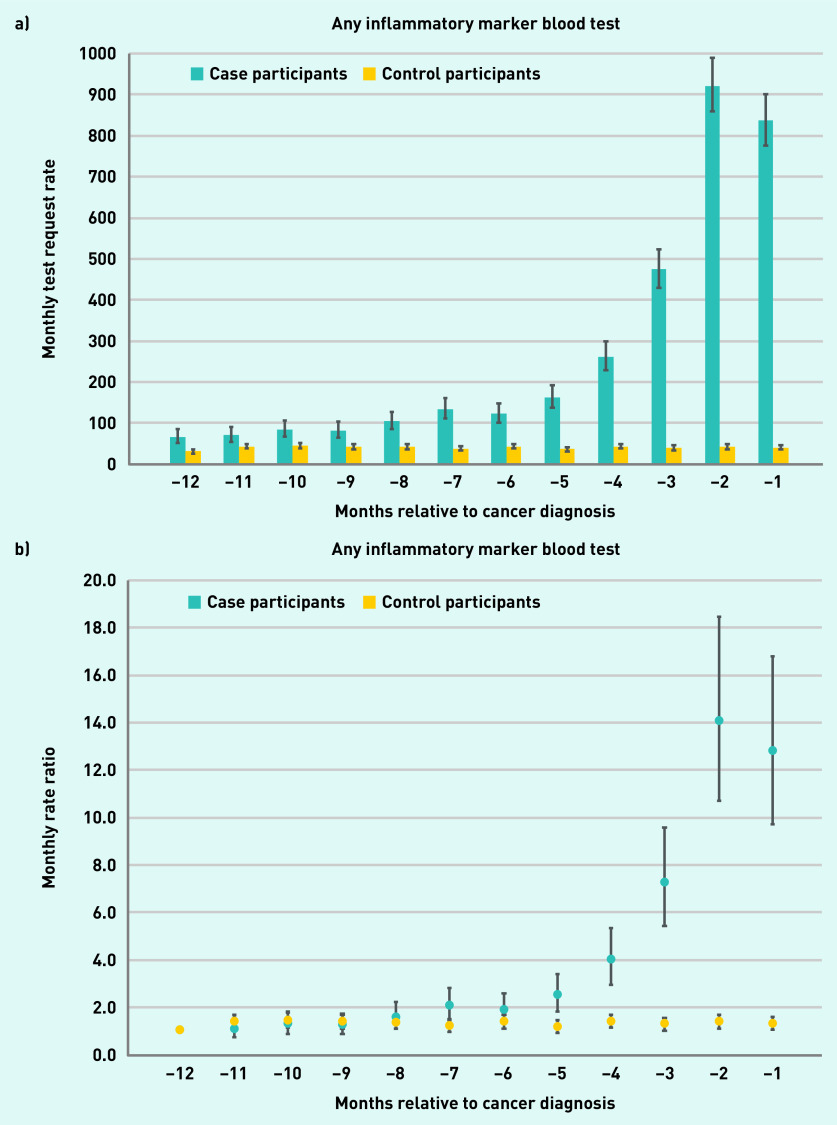
*GP requests for any inflammatory marker blood test in patients with Hodgkin lymphoma and controls. a) Rate of blood test requests (test requests per 1000 patients per month) 12 months before diagnosis/index date with 95% CIs. b) The rate ratio for test requests compared with baseline at −12 months with 95% CIs. CI = confidence interval.*

Among patients with Hodgkin lymphoma, the rate of inflammatory marker requests increased throughout the year pre-diagnosis, with monthly testing rates increasing 13-fold from 66 tests per 1000 patients at baseline to 836 tests per 1000 patients in the month immediately before diagnosis (RR 12.8, 95% CI = 9.7 to 16.8, *P*<0.001), while remaining stable in controls over the same period (Figure 2 and Supplementary Table S5).

**Figure 3.**
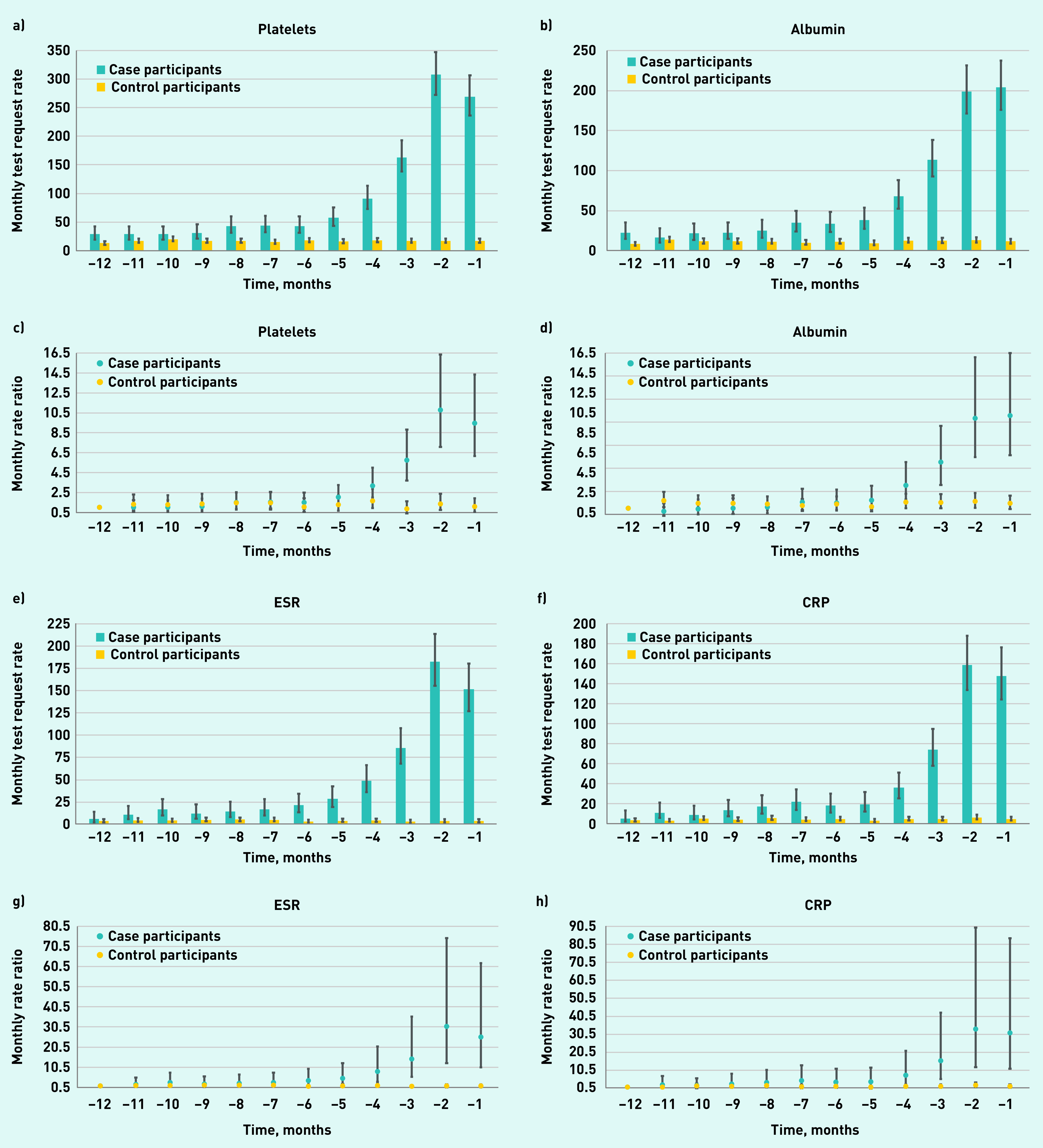

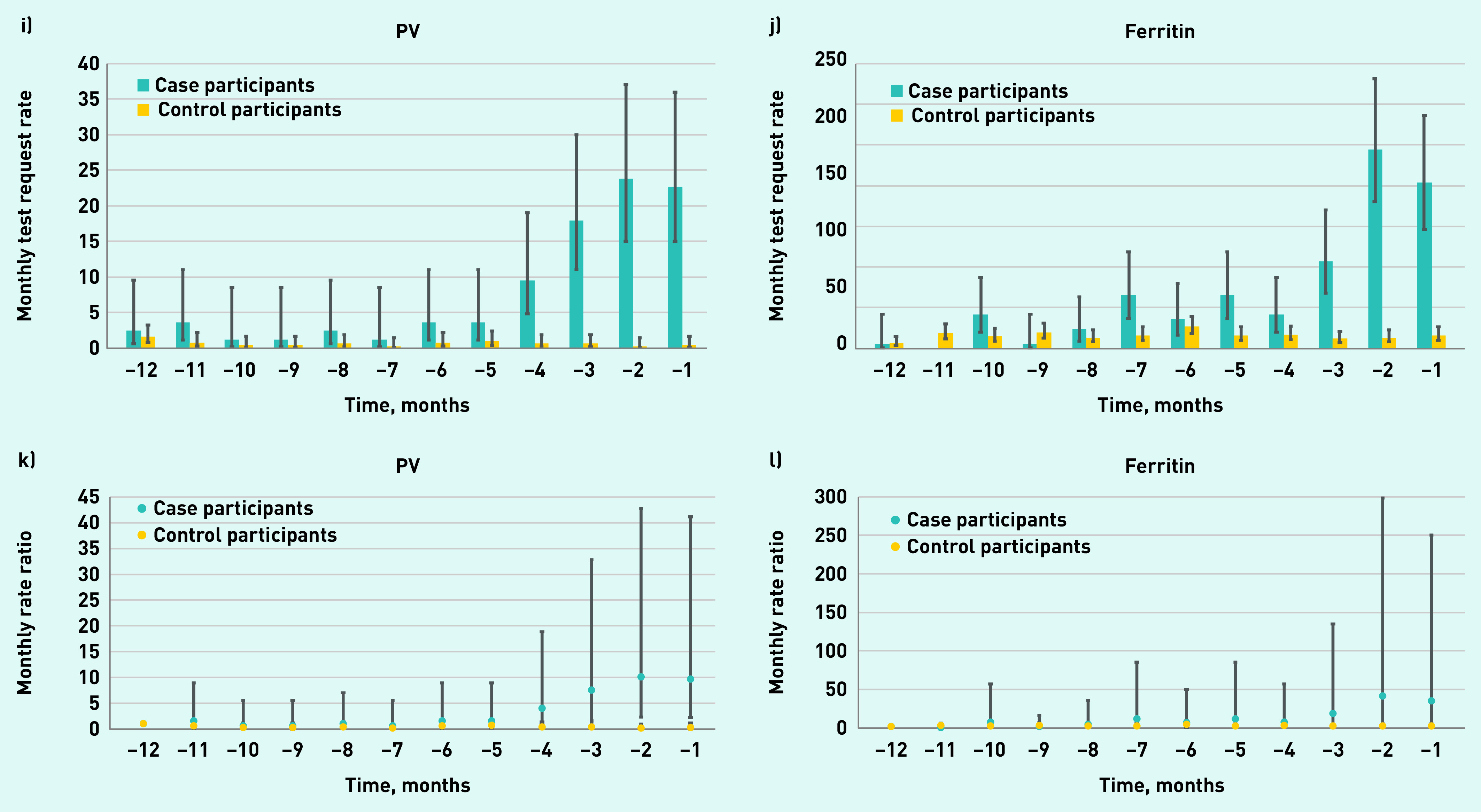
*GP requests for inflammatory marker blood test in patients with Hodgkin lymphoma and controls by test type. a) and c) platelets; b) and d) albumin; e) and g) ESR; f) and h) CRP; i) and k) PV; and j) and l) ferritin. a), b), e), f), i), and j) = rates of blood test requests (test requests per 1000 patients per month) 12 months before diagnosis/index date with 95% CIs. c), d), g), h), k), and l) = the rate ratio for test requests compared with baseline at −12 months with 95% CIs. CI = confidence interval. CRP = C-reactive protein. ESR = erythrocyte sedimentation rate. PV = plasma viscosity.*

Considering the use of different inflammatory marker tests individually over time, among patients with Hodgkin lymphoma, an increase in GP test request rates was apparent for each of the six inflammatory marker tests throughout the year pre-diagnosis (Figure 3).

### Inflammatory marker test results over time

**Figure 4.**
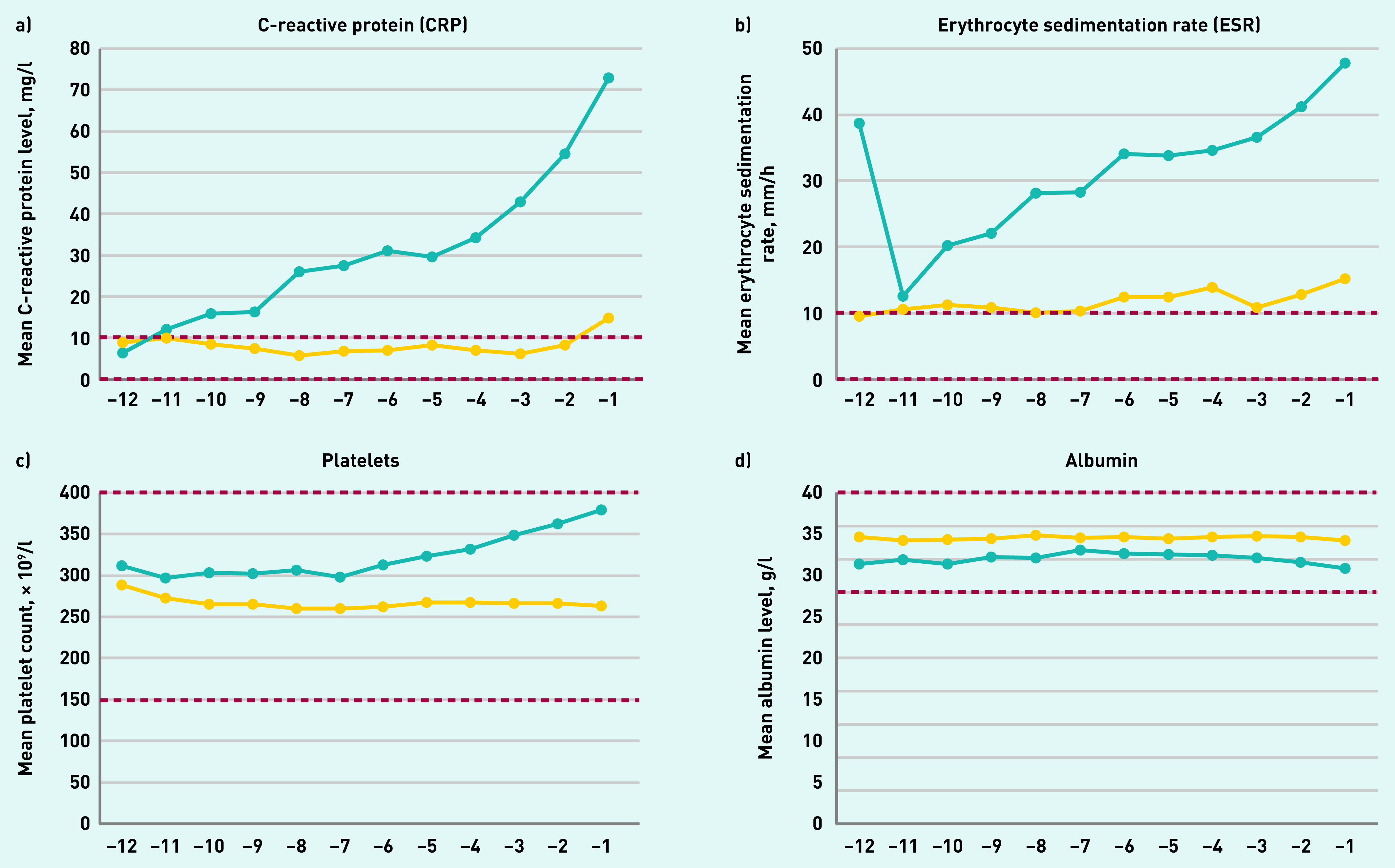
*Inflammatory marker blood test trajectories 12 months before diagnosis for the four most commonly requested tests — mean test value per month using a 3-month moving average in general practice for tested patients with Hodgkin lymphoma patients (blue line) and controls (yellow line). Dashed line represents upper and lower limit of normal range.*

Among tested patients, trends over time in inflammatory marker levels of the four most commonly requested inflammatory markers with large enough number of observations showed that mean monthly values of ESR and platelet levels were consistently higher in patients with Hodgkin lymphoma for all 12 months pre-diagnosis and from 11 months pre-diagnosis for CRP. Mean albumin levels were consistently lower than in controls for all 12 months pre-diagnosis (Figure 4).

While remaining stable in controls, among patients with Hodgkin lymphoma the mean CRP levels increased throughout the year before diagnosis, mean ESR levels from 11 months pre-diagnosis, and mean platelet levels from around 7 months pre-diagnosis.

### Co-occurrence of inflammatory marker test use with red-flag symptoms

Among all inflammatory marker-tested patients with Hodgkin lymphoma, 39.4% (234/594) had no ‘red-flag’ symptoms recorded in the year before diagnosis (Table 2 and Supplementary Table S6). Of the 413 inflammatory marker-tested patients with Hodgkin lymphoma with an abnormal result 42.6% (176/413) had no red-flag symptoms recorded (see Supplementary Table S6).

**Table 2.** The frequency of red-flag symptoms in patients with Hodgkin lymphoma who had inflammatory marker tests (*n* = 594)

**Symptom**	**Platelet, *n* (%) (*n*= 578)**	**Albumin, *n* (%) (*n* = 452)**	**ESR, *n* (%) (*n*= 361)**	**CRP, *n* (%) (*n*= 320)**	**Ferritin, *n* (%) (*n* = 113)**	**PV, *n* (%) (*n*= 51)**
No red-flag symptoms	226 (39.1)	187 (41.4)	142 (39.3)	137 (42.8)	64 (56.6)	16 (31.4)
Lymphadenopathy	167 (28.9)	118 (26.1)	99 (27.4)	81 (25.3)	24 (21.2)	18 (35.3)
Lump^a^	211 (36.5)	155 (34.3)	128 (35.5)	111 (34.7)	23 (20.4)	27 (52.9)
Night sweats	26 (4.5)	23 (5.1)	19 (5.3)	20 (6.3)	6 (5.3)	1 (2.0)
Weight loss	25 (4.3)	21 (4.6)	16 (4.4)	15 (4.7)	5 (4.4)	4 (7.8)

a *Lump is defined as in the head/neck, axillary, groin, or not otherwise specified. CRP = C-reactive protein. ESR = erythrocyte sedimentation rate. PV = plasma viscosity.*

Similarly, among all tested patients with Hodgkin lymphoma without red-flag symptoms, 75.2% (176/234) had at least one abnormal result and 25.2% (59/234) had an ‘early’ abnormal result (data not shown).

## DISCUSSION

### Summary

In patients with Hodgkin lymphoma, both GP requests for inflammatory marker blood tests and inflammatory marker levels increase throughout the year before diagnosis when compared with controls. Studies investigating aetiological associations between markers of inflammation and Hodgkin lymphoma should exclude the year pre-diagnosis to avoid reverse causation. These increases also represent early signals of disease and indicate that a ‘diagnostic time window’ of appreciable length exists for earlier diagnosis of Hodgkin lymphoma in at least some patients.

Over 70% of all patients with Hodgkin lymphoma had at least one inflammatory marker blood test in the year before diagnosis, with two-thirds of these patients having at least one abnormal result and one in five having an ‘early’ abnormal result ≥3 months before their diagnosis. Close to half of all patients with Hodgkin lymphoma with an abnormal result had no other ‘red-flag’ features recorded. Inflammatory marker tests may therefore provide information that could support earlier diagnosis in large proportions of patients with Hodgkin lymphoma presenting with non-specific symptoms to primary care, if enabled by advances in diagnostic processes and technologies.

### Strengths and limitations

This UK nationwide study is, to the best of the authors’ knowledge, the first to explore patterns of inflammatory marker tests in primary care over time before a diagnosis of Hodgkin lymphoma, together with consideration of whether abnormal results occurred in patients presenting with or without ‘red-flag’ symptoms. Strengths include the large sample size, which is representative of the UK population,^19^ and its primary care setting; these aspects increase the generalisability of the findings. Test request rates were plotted alongside monthly RRs to determine the diagnostic time window length. The recording of cases of lymphoma in CPRD concords highly with English population-based cancer registration data,^25^ and was further enhanced by linkage to hospital records.^26^ Because blood results are electronically incorporated into patients’ primary care records the likelihood of inaccuracies is reduced. However, in a relatively small proportion of patients, blood tests may have been requested by their GP but not completed and therefore not coded. In some patients a ‘red-flag’ symptom may have been present but not coded, resulting in possible underestimation of ‘red-flag’ feature frequency; however, given the clinical importance of these symptoms and high awareness among GPs such under-coding is likely rare.^27^

The current study included patients with Hodgkin lymphoma ≤50 years. Because Hodgkin lymphoma subtypes and their association with inflammation are likely to differ by age,^8^^,^^9^^,^^28^^–^^34^ findings are not necessarily generalisable to the diagnostic pathway of older patients with Hodgkin lymphoma. It was not possible to assess for overdispersion; therefore, if present the confidence intervals presented may be somewhat narrower than they should be. A 12-month pre-diagnosis period was used in this study, guided by prior epidemiological studies indicating that the length of this period is adequate to assess reverse causality from medication prescriptions and cancer incidence.^16^ However, in the current study it was not possible to confirm if this also applies for the association between inflammatory markers and Hodgkin lymphoma. Larger future studies should examine longer pre-diagnostic periods and additional blood tests to find out if similar patterns are seen.

### Comparison with existing literature

A few studies have investigated the association between primary care inflammatory marker tests and subsequent cancer diagnosis. These have shown that raised platelets,^35^^,^^36^ ESR, CRP, and PV,^37^^,^^38^ and hypoalbuminaemia^39^ are risk factors for undiagnosed cancer of any site within the next year, particularly in patients with persistent and/or greater inflammatory marker abnormalities, or ≥2 abnormal inflammatory marker test results.^37^^,^^38^ Raised inflammatory markers (platelets, ESR, CRP, or PV) are associated with increased risk of subsequent Hodgkin lymphoma diagnosis in patients aged ≥40 years with red-flag symptoms.^11^ Studies of other cancer sites have reported raised CRP levels to be predictive of lung cancer up to 12 months before diagnosis;^18^ and increases in ESR and PV levels up to 2 years before a myeloma diagnosis.^17^

The current study adds to these findings by demonstrating for the first time that similar phenomena exist for Hodgkin lymphoma, profiling additional inflammatory marker blood tests, demonstrating the value of abnormal inflammatory results in patients without ‘red-flag’ symptoms, and by determining the length of the lag-period that should be applied to future aetiological studies examining inflammation and Hodgkin lymphoma development.

### Implications for research and practice

Increases in inflammatory marker levels in patients with Hodgkin lymphoma were concentrated in the months leading up to diagnosis, which strongly suggests that they are a result of the evolving neoplastic process^12^ rather than related to pre-existing chronic inflammatory conditions. Therefore, a minimum of 12-months pre-diagnosis should be excluded in studies examining aetiological associations between inflammatory markers and Hodgkin lymphoma risk to ensure associations do not reflect underlying malignancy.

The increased rate of inflammatory marker requests during the year before Hodgkin lymphoma diagnosis suggests many patients with Hodgkin lymphoma are presenting to their GP several months pre-diagnosis with symptoms prompting further investigation and the presence of an appreciable ‘diagnostic time window’ for potential earlier diagnosis in some patients with Hodgkin lymphoma if this increased activity can be detected.

Patients with Hodgkin lymphoma frequently have abnormal results several months pre-diagnosis, often in the absence of red-flag symptoms such as lymphadenopathy. This indicates that an inflammatory response is also occurring in patients with Hodgkin lymphoma with non-specific symptoms, and abnormal inflammatory marker levels can represent early detectable signs of Hodgkin lymphoma in this group. As such abnormalities are relatively common in primary care and Hodgkin lymphoma is a rare disease, the predictive value for Hodgkin lymphoma of any such single result in isolation will likely be low. However, if supported by advances in diagnostic processes and technologies, there is potential for evidence from blood test results to be combined with other pre-diagnostic features to provide information that could support earlier Hodgkin lymphoma diagnosis in some patients.

## Data

Raw data are not publicly available but are available to researchers via application to the CPRD.
